# Research of thermal sensor allocation and placement based on dual clustering for microprocessors

**DOI:** 10.1186/2193-1801-2-253

**Published:** 2013-06-04

**Authors:** Xin Li, Mengtian Rong, Tao Liu, Liang Zhou

**Affiliations:** Key Laboratory of Ministry of Education of China for Research of Design and Electromagnetic Compatibility of High Speed Electronic Systems, Shanghai Jiao Tong University, Shanghai, 200240 China

**Keywords:** Dynamic thermal management, Thermal sensors, Allocation, Placement, Dual clustering, Thermal gradient

## Abstract

Dynamic thermal management techniques employ a set of on-chip thermal sensors to measure runtime thermal behavior of microprocessors so as to prevent the on-set of high temperatures. Therefore, effective analysis of thermal behavior and determination of the best allocation and placement of thermal sensors directly impact the effectiveness of the dynamic thermal management mechanisms. In this paper, we propose systematic and effective techniques for determining the fewest number of thermal sensors and the optimal locations based on dual clustering to provide a high fidelity thermal monitoring. Initially, we utilize the dual clustering algorithm to devise method that can reduce the number of sensors to a great extent while satisfying an expected accuracy. Then we identify an optimal physical location for each sensor such that the sensor’s attraction towards steep thermal gradient is maximized. Experimental results indicate the superiority of our techniques and confirm that our proposed methods are capable of creating a sensor distribution for a given microprocessor architecture using the number of thermal sensors of 2, 8, 15, 24, 35, depending on different expected hot spot temperature error accuracy of 5%, 4%, 3%, 2%, 1%, respectively.

## Introduction

Large-scale circuit integration and exponentially increasing power densities have resulted in high temperature in current microprocessors. Elevated chip temperature slows down transistor speed and increases interconnect delays (Brooks et al. 2007). The results of these trends are timing failures and thermal runaway (Lin & Banerjee 2008). Therefore, effective assessment and analysis of the thermal behavior of microprocessors have become a major issue to be considered.

Traditionally, the problem of temperatures on chips has been solved by employing dynamic thermal management techniques (Jayaseelan & Mitra 2009) which use a set of on-chip thermal sensors that continuously monitor temperatures at a few selected die locations during the runtime. The most well-known dynamic thermal management techniques include clock gating, dynamic voltage and frequency scaling (DVFS) (Hanson et al. 2007). Several microprocessors have been equipped with thermal sensors. For instance, AMD Opteron employs 38 thermal sensors (Zhang & Srivastava 2009; Zhang & Srivastava 2011) that trigger alarms if the junction temperature exceeds a specified limit (Coskun et al. 2008).

Moreover, accuracy is another crucial criterion for dynamic thermal management techniques. Overestimation of temperature results in spurious alerts that lead to unnecessary triggering of thermal control mechanisms, e.g., DVFS (Long et al. 2008; Memik et al. 2008). On the other hand, underestimation of temperature greatly reduces the reliability since the processor will continue to operate at a higher temperature than its rated operating condition (Long et al. 2008; Memik et al. 2008). Embedding a large number of thermal sensors on the die is an unadvisable option to increase the accuracy. In fact, chips need to use the fewest number of thermal sensors to reduce manufacturing costs, die area and design complexity. In addition, allocating arbitrarily large number of sensors employed by the monitoring infrastructure, constructing the sensor networks will also pose a challenge (Long et al. 2008). An ideal goal is to monitor the highest temperatures on a microprocessor with allocating a minimum number of thermal sensors. As a result, how to provide accurate thermal monitoring in a given system while maintaining a reasonable number of sensors becomes crucial.

In this paper, we propose systematic and effective techniques for determining the fewest number of thermal sensors and the optimal locations based on dual clustering algorithm to provide a high fidelity thermal monitoring.

The organization of this paper is as follows. Related Work section overviews some of the recent relevant methods in the literature. In Proposed Thermal Sensor Allocation and Placement Techniques section we provide an overview of our methodology, where we introduce the thermal gradient calculation method in Thermal Gradient Calculation section and propose effective technique for thermal sensor allocation based on the dual clustering algorithm in Sensor Allocation Scheme section, and in Sensor Placement Strategies section we identify an optimal strategy for thermal sensor placement. We demonstrate the effectiveness of our methods through an extensive set of experimental results in Experimental Results section. Finally, Conclusion section summarizes the main conclusions of this work and indicates directions for future work.

## Related work

It is intriguing to observe that several recent studies aiming to address thermal sensor allocation problem and reconstruct the full thermal characterization seemed to have a few works. For thermal sensor allocation and full thermal reconstruction, some representative techniques have been proposed shown in Table 1.

**Table 1:** **Overview of related works**

Motivation	Reference	Methodology
Thermal sensor allocation	Long *et al*. (2008)	• Grid-based interpolation scheme
	Memik *et al*. (2008)	• Uniform allocation: interpolation scheme
		• Non-uniform allocation: improved *k*-means clustering algorithm
	Nowroz *et al*. (2010)	• Min-cut placement techniques: recursively allocating the sensors to the different die regions depending on their spectral energy
	Reda *et al*. (2011)	• Hard sensor allocation techniques: Heuristic iterative approach to approximate an NP-hard problem
		• Soft sensor computation techniques: a weighted linear combinations of the measurements of the hard sensors
Full thermal reconstruction	Cochran *et al*. (2009)	• Spectral techniques
	Li *et al*. (2011)	• Inverse distance weighting method based on a dynamic Voronoi diagram

## Proposed thermal sensor allocation and placement techniques

Although it is a clear trend in elevating the number of thermal sensors in high performance microprocessors (Long et al. 2008), allocating the number of sensors arbitrarily will create several overheads as mentioned earlier. Reducing the number of sensors may help relieve these overheads. However, this will cause inaccuracies. Our goal is to provide accurate thermal monitoring while maintaining a reasonable number of sensors. In this section we first introduce the thermal gradient calculation method, and then we propose systematic and effective thermal sensor allocation and placement techniques to overcome this challenge.

### Thermal gradient calculation

Thermal gradient describes that in which direction and at what rate the temperature changes the most rapidly around a particular location. The magnitude of the thermal gradient determines how fast the temperature changes in the corresponding direction rather than the value of the temperature at the measuring point.

Any representation in computer memory must be discretized, we utilize the classical Sobel operator (Wang 2009) to calculate an approximation of the gradient of the thermal map. At each point in the thermal map, the result of the Sobel operator is either the corresponding gradient vector or the norm of this vector. The Sobel operator is implemented using the following two 3 × 3 matrixes which are convolved with the original thermal map to calculate approximations of the derivatives: one for horizontal changes, and the other for vertical.1

**Figure 1:**
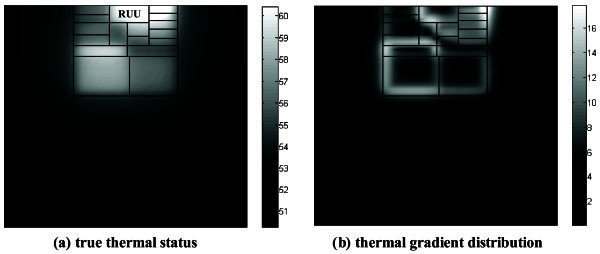
**Thermal gradient calculation for bizp2 (Henning**^2000^**). a**: true thermal status; **b**: thermal gradient distribution.

If we define *T* as the source thermal map, at each point in the thermal map, the approximation of the magnitude of the thermal gradient is expressed as follows:2

where ‘*’ here denotes the *2*-dimensional convolution operation.

(Memik *et al*^2008^) indicated that the thermal gradient around a high-temperature location is larger than that at a low-temperature point. However, our experimental results find that the thermal gradient at one point has no relation with its own temperature. For example, we simulated the bzip2 benchmark (Henning 2000) using the experimental flow shown in Simulation Infrastructure section. Figure 1 (a) exhibits the full thermal characterization, and Figure 1 (b) shows the thermal gradient distribution calculated by Sobel operator. It’s observed that the RUU block has relatively high temperatures, while attaining lower values in thermal gradient distribution.

### Sensor allocation scheme

In general, placing sensors at the hot spot locations for one application will cause large temperature errors for other applications (Memik et al. 2008; Mukherjee & Memik 2006). Our objective is to address this deficiency by systematically analysis of thermal maps across a wide set of applications. We formulate the sensor allocation problem as a dual clustering of the points of interest in the spatial and non-spatial domains. We try to partition the hot spot data set into several groups, so that these groups form nonoverlapping compact regions in the spatial domain while minimizing the dissimilarity of the data points in a group on the non-spatial domain (Lin et al. 2005). Then, each group will be allocated one sensor, which will monitor the hot spot points associated with that group. In the remaining part of this section, we will first briefly introduce the basic concept of the dual clustering. Based on the dual clustering, we propose an effective sensor allocation algorithm.

#### Dual clustering

The dual clustering (Jiao et al. 2011) can be defined as: given a set of objects {*o*_1_, *o*_2_, …, *o*_*n*_}, each object has two attribute domains, i.e., spatial domain and non-spatial domain, as shown in Equation 3.3

Where  is the spatial location (*L* is usually set to 1, 2 or 3), and  is the non-spatial attributes (*T* is the number of non-spatial attributes). The spatial distance between two objects is defined as Euclidean distance, and the non-spatial distance between two objects is given by Equation 4.4

Where  is the non-spatial distance between object *i* and object *j*,  and  represent the values of attribute *t* for object *i* and object *j*, *w*_*t*_ is the weight of attribute *t*, and .

Dual clustering is the process of partitioning the object data set into several groups, while clustering dispersion in the non-spatial domain is less than the given threshold and each group is a connective cluster (Jiao et al. 2011). The result of dual clustering should be spatial continuous and attributively aggregative.

#### Sensor allocation algorithm

**Figure 2:**
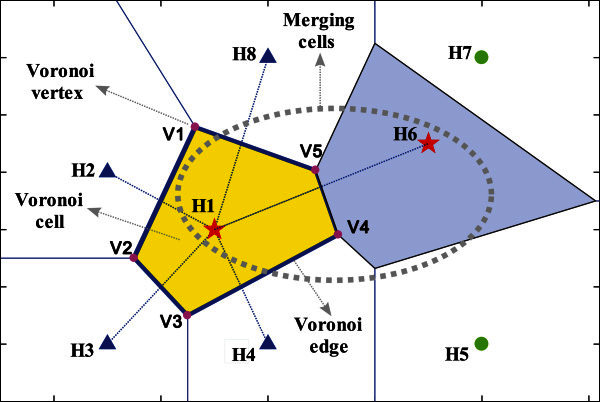
**Illustration of using Voronoi diagram to detect and merge adjacent cells.**

Based on dual clustering, we devise an effective sensor allocation algorithm. Initially, we construct a Voronoi diagram (Bhattacharya & Gavrilova 2007) according to the locations of all the hot spots on the die. After that, the hot spot fields are divided into subregions of Voronoi cells, and each stationary hot spot node is within a Voronoi cell shown in Figure 2.

- **Definition 1.** If the Voronoi cells of two hot spots share a Voronoi edge (have more than a single point in common), then the two hot spots are considered neighbor, i.e., the hot spots H2, H3, H4, H6 and H8 are Voronoi neighbors of hot spot H1 in Figure 2.
- **Definition 2.** Set the number of non-spatial attributes *T* to 1 and the non-spatial attribute is defined as the temperature of hot spot.
- **Definition 3.** If two hot spots are neighbor to each other and the non-spatial distance between them is less than the given threshold , then the Voronoi cells of the two hot spots are merged into a new cluster, i.e., in Figure 2, the Voronoi cells of hot spot H1 and H6 are merged into a cluster when .
- **Definition 4.** Setthethresholdofnon-spatialdistanceto:
  5

Where *α* is a correction coefficient, *ϵ*_max_ is an expected hot spot temperature error accuracy, *n* is the number of hot spots in a cluster and *a*_*i*_ is the value of non-spatial attribute at each hot spot in a cluster.

Our sensor allocation algorithm can be presented as follows:

1. Select a hot spot with maximum value of thermal gradient as initial cluster center.
2. Apply the definition 3 to obtain a new cluster *C*_*new*_.
3. Set the hot spots in cluster *C*_*new*_ to new cluster centers and go to Step 2.
4. If the cluster *C*_*new*_ cannot be merged with other cells, it is defined as an integrated cluster, then allocate one sensor to it.
5. Perform the Step 1–4 in residual hot spots until each hot spot belong to a certain cluster.

**Figure 3:**
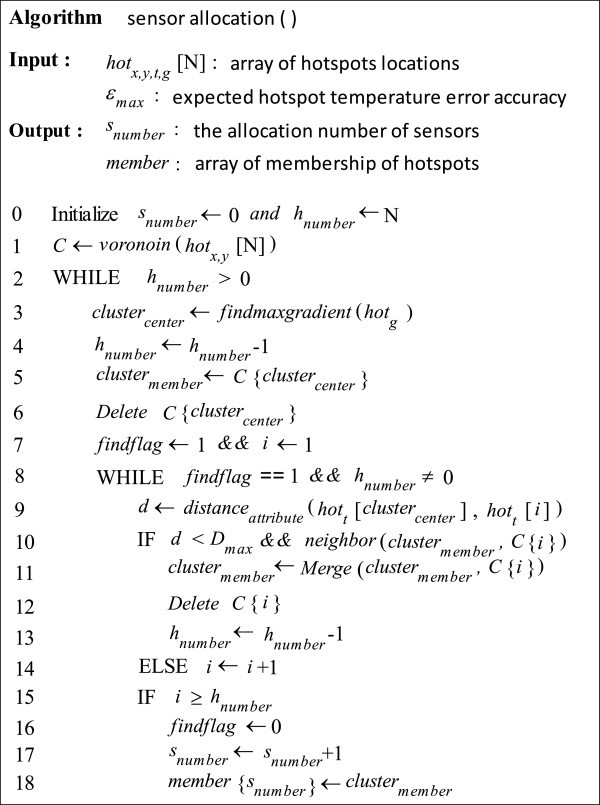
**Pseudocode for the sensor allocation algorithm.**

The details of sensor allocation algorithm are shown in Figure 3.

### Sensor placement strategies

Once we finish the hot spot clustering, the allocation number of sensors is determined. Then we need to determine the physical location of thermal sensors. In this section we identify two different strategies for thermal sensor placement.

- Geometric-Center Sensor Placement. In this strategy, a sensor is placed at the geometric center of each cluster region.

As we know, ideal thermal sensor placement methods that focus on placing sensors only near potential locations which have the highest absolute temperatures will achieve the best results for hot spot temperature estimation. However, these methods might lead to poor results for full thermal reconstruction as they will have no information at the locations which temperatures change the most rapidly. Thus, we choose thermal gradient, instead of absolute temperature, as the base for sensor placement method. We propose here another strategy-which is inspired by improved *k*-means clustering method (Memik et al. 2008; Mukherjee & Memik 2006)-that takes into account the diversity of thermal gradients within a cluster.

- Thermal-Gradient-Attraction Sensor Placement. The basic idea behind this strategy is to move the sensors closer to the relatively higher thermal gradient hot spots. This is equivalent to the sensor being attracted to the hot spots with high thermal gradient values by a larger force (Memik et al. 2008; Mukherjee & Memik 2006). The details of this strategy are described as follows:

For each addition of hot spot *h*_*i*_ of cluster *C*_*j*_, the sensor coordinates are the cumulative sum of the corresponding member coordinates. The cumulative sum computation is shown in Equation 6.6

**Figure 4:**
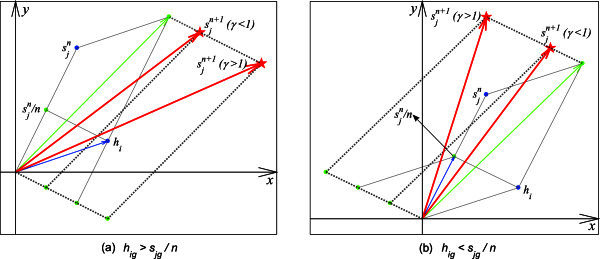
**Illustration for the thermal gradient attraction approach. a**: when *h*_*ig*_ is greater than *s*_*jg*_/*n*; **b**: when *h*_*ig*_ is less than *s*_*jg*_/*n*.

Where *s*_*jx*,*y*_, *h*_*ix*,*y*_ and *s*_*jg*_, *h*_*ig*_ are the coordinates and thermal gradient of sensor *s*_*j*_ and hot spot *h*_*i*_, respectively. *n*_*iteration*_ is the number of iterations, and *β* is an attraction coefficient. We have determined experimentally that an attraction coefficient value *β* = 0.3 performs best. The (*x*, *y*) coordinates of the sensor *s*_*j*_ are closer to the hot spot *h*_*i*_ if the *g* dimension of *s*_*j*_ is less than that of *h*_*i*_, otherwise the sensor moves further from the position of *h*_*i*_. The illustration for the thermal gradient attraction approach in the *n* + 1 iteration is shown in Figure 4 (*γ* = *β*(*h*_*ig*_ − *s*_*jg*_/*n*_*iteration*_)). After iterating over all the hot spots in cluster *C*_*j*_, the final position of *s*_*j*_ is updated as shown in Equation 7.7

## Experimental results

In the following two sections we first describe our experimental methodology and then we present our results.

### Simulation infrastructure

To evaluate the effectiveness of our methods, we design an experimental flow that simulates thermal distribution for a 65 nm microprocessor based on Alpha EV6 architecture. We first give the definition of power consumption (Shauly 2012) and then we describe our experimental flow.

#### Power consumption

There are two main components that constitute the power used by a CMOS integrated circuit: static power and dynamic power. Static power essentially consists of the power used when the transistor is not in the process of switching. Typically, CMOS technology has been praised for its low static power. However, as devices are scaled, gate oxide thicknesses decrease and there is increased probability of tunnelling, resulting in larger and larger leakage currents. Therefore, static power (also called leakage power) dissipation will become increasingly significant. Dynamic power is the sum of transient power consumption and capacitive load power consumption. The total power dissipation is summarized as shown in Equation 8:8

*P*_*short*_ is the power consumed during gate voltage transient time, that in CMOS technology is only related to the direct path short circuit current (*I*_*SC*_) which flows when both the NMOS and PMOS transistors are simultaneously active, conducting current directly from supply to ground. Significant short circuit power dissipation can be avoided if the output rise/fall time of a gate is much longer than the input rise/fall time. *P*_*switch*_ refers to the dynamic component of power, where *C*_*L*_ is the total loading capacitance, *f* is the clock frequency, and *ψ* is the average switching activity factor. *P*_*static*_ is due to the leakage current *I*_*leakage*_. Imperfect cut-off of the transistor leads to leakage (*I*_*leakage*_) and power dissipation (*P*_*static*_) even without any switching activity.

#### Experimental flow

**Figure 5:**
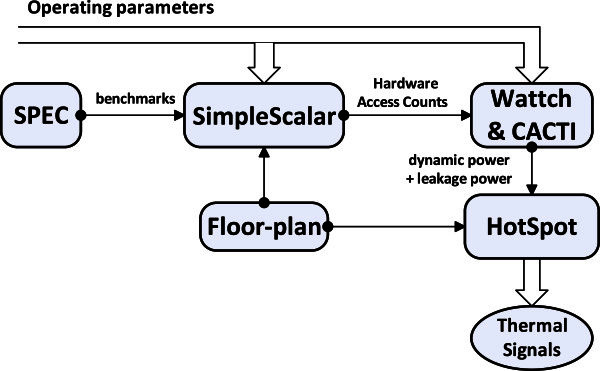
**Experimental flow for simulating thermal distribution.**

**Figure 6:**
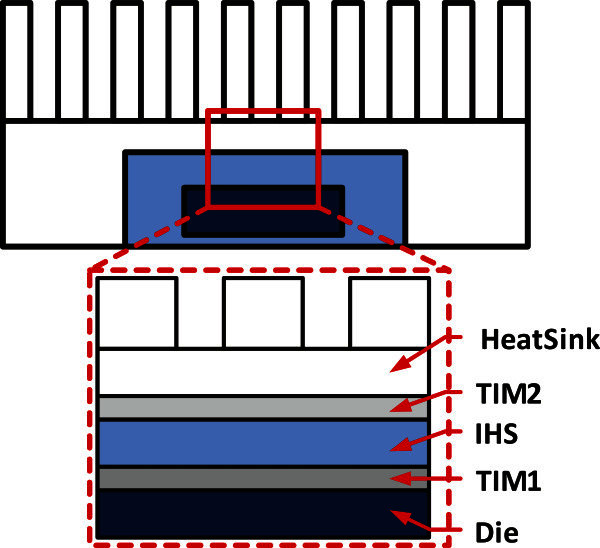
**Sketch of a microprocessor package assembly.**

The complete experimental flow shown in Figure 5 is performed using the following tools:

- We use the Alpha EV6 as our base processor (Kessler 1999) with a 3 GHz clock frequency. The Alpha EV6 is an out-of-order speculative execution core that is commonly used as a test-bench core in thermal management research.
- For workloads, we simulated the SPEC2000 benchmark (13 floating points and 12 integer benchmarks) suite (Henning 2000), using Simple Scalar (Burger & Austin 1997) 3.0e. The Simple Scalar simulates a superscalar processor with out-of-order issue and execution. For each application, we simulated 10 million instructions.
- For dynamic power estimation, we use Wattch (Brooks et al. 2000), a power simulator for analyzing and calculating microprocessor power dissipation at the architecture-level. We integrate the Wattch power model into Simple Scalar simulator in order to gain the power statistics in each time interval. For each functional unit in the processor, we add an access counter to record the access information, which is fed into the Wattch power model to calculate the dynamic power traces. In our experiments, we assume clock gating to all components and that clock gating can reduce dynamic power by 75%, as proposed by Liao *et al*. (2005). For leakage power estimation of processor core units, we construct a leakage model (Liao et al. 2005) and use CACTI 5.0 (Wilton & Jouppi 1996) to accurately model cache leakage power.
- We utilize HotSpot (Huang et al. 2006) version 5.0 for thermal simulation in the grid level (discretized into 128 × 128 grids). The floor-plan of Alpha EV6 and the workload power traces from Wattch are used as inputs to the HotSpot, and finally the steady-state temperatures for a set of grid locations can be produced as output. This type of grid level thermal modelling is useful for capturing spatial temperature variation within a processor unit. The initial temperature of processor, which represents the die temperature if the processor was already executing instructions prior to execution of benchmarks to model the warm up period, was assumed to be 60*°C*. The ambient temperature is set to 45*°C*. For 3GHz clock frequency, HotSpot calling interval of 10 K cycles gives the best trade-off between precision and overhead (Mukherjee & Memik 2006). The package assembly model in HotSpot, whose physical and thermal properties of all packaging layers are evaluated according to a practical packaged high-performance microprocessor shown in Table 2 (Lin et al. 2007), was also created shown in Figure 6.

**Table 2:** **Dimensions and thermal properties of different package layers**

Layer	Area (***mm***^2^)	Thickness ( ***mm*** )	Mesh length ( ***mm*** )	Specific heat ( ***J*** / ***kg°C*** )	Density ( ***kg*** /***m***^3^)	Thermal conductivity ( ***W*** / ***m°C*** )
Die	10 × 10	0.8	0.08	712	2330	148
TIM1	10 × 10	0.4	0.08	230	7310	30
IHS	30 × 30	2.4	0.2	385	8930	390
TIM2	30 × 30	0.4	0.2	2890	900	6.4
HeatSink	60 × 60	6.4	0.4	385	8930	360

**Figure 7:**
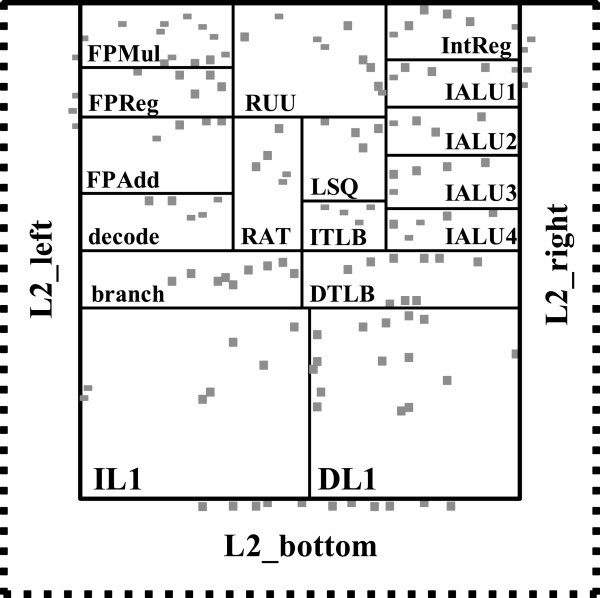
**Distribution of the hot spots (marked with squares) for each processor block for SPEC2000 benchmarks.**

The point of interest for our experiments is the hottest point per component. For each benchmark, each component will exhibit a hot spot. As the location of this hot spot may change for different applications as verified by Memik *et al*. (2008), we first combine these locations to find the distribution of hot spots across different benchmarks. Figure 7 depicts the distribution of hot spots for each processor block, which was obtained from our simulations across the SPEC2000 benchmarks in the same Alpha EV6 architecture. Dotted lines represent the region of L2 cache blocks containing the hot spots. We partition the L2 cache into three regions: L2_left, L2_right and L2_bottom. Across 25 benchmarks and 20 different components of the processor, the theoretical number of block-level hot spots is 500. However, some hot spots reoccur due to correlation of activity and power density, and the temperatures of some hot spots are obviously lower than those of other hot spots in the same block, leaving us with 132 distinct points. Based on this distribution we make decisions of the allocation number and locations of sensors using our proposed thermal sensor allocation and placement techniques as described in Proposed Thermal Sensor Allocation and Placement Techniques section.

## Results

Extensive experiments are conducted to examine the effectiveness of our proposed thermal sensor allocation and placement techniques. All experiments are implemented by MATLAB code and run on a Pentium 3.0 GHz PC with 1GB SDRAM. In our experiments we report the following three metrics:

- Number of thermal sensors. Given a maximum allowable hot spot temperature error accuracy: for our proposed thermal sensor allocation and placement techniques, we determine the number of integrated cluster and each integrated cluster will be allocated one sensor; for improved *k*-means clustering technique (Memik et al. 2008), we iteratively perform the improved *k*-means clustering algorithm until the maximum hot spot estimation error is less than the given allowable hot spot temperature error (initially, set the value of *k* to 1). Finally, the value of *k* is the number of thermal sensors.
- Hot spot estimation error. The computation of the hot spot estimation error is equal to the difference between the hot spot temperatures in the true temperature distribution signals as obtained by executing the experimental flow and the temperatures at the locations of the thermal sensors.
- Full thermal reconstruction error. For each application, we reconstruct the full thermal characterization with the different strategies for thermal sensor placement, using the inverse distance weighting method based on a dynamic Voronoi diagram (Li et al. 2011). Then, we compute the average absolute temperature error between the true temperatures and the estimated temperatures calculated by the reconstruction method. We report the average absolute error computed for all 25 benchmarks.

**Figure 8:**
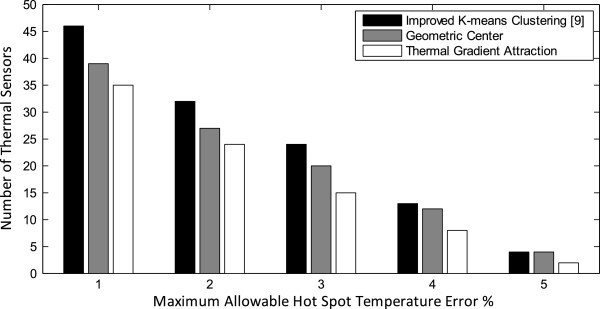
**Allocation number of thermal sensors using various sensor allocation and placement methods.**

In our first set of experiments, we determine the allocation number of thermal sensors while varying the maximum allowable hot spot temperature error accuracy from 1% to 5%. We compare three different methods for thermal sensor allocation and placement: improved *k*-means clustering (IKmC) (Memik et al. 2008), geometric-center (GC) and thermal-gradient-attraction (TGA). The plot in Figure 8 gives the allocation number of thermal sensors while satisfying different maximum allowable hot spot temperature error accuracy. Comparing the results, it’s observed that our proposed thermal gradient attraction method gives the fewest number of thermal sensors and all of our proposed methods significantly outperform the improved *k*-means clustering (Memik et al. 2008). Allocating arbitrarily large number of sensors will not only create a significant area overhead, but constructing the sensor networks will also pose a challenge. Thus, reduce the number of thermal sensors to a great extent while satisfying the maximum allowable hot spot temperature error accuracy is a desirable property for microprocessors.

**Figure 9:**
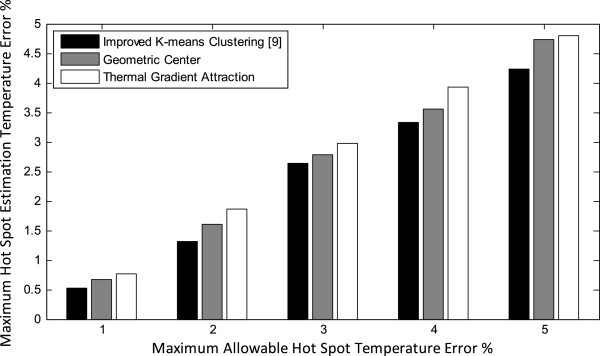
**Maximum hot spot estimation temperature error using various sensor allocation and placement methods.**

In the second set of experiments we demonstrate that the maximum hot spot estimation temperature error obtained by our proposed sensor allocation and placement techniques is assuredly less than the corresponding maximum allowable hot spot temperature error. We repeat this experiment using different maximum allowable hot spot temperature errors and report the errors in hot spot estimation in Figure 9. The results are summarized in Table 3. The results show that our proposed sensor allocation and placement methods give close results to improved *k*-means clustering (Memik et al. 2008), while reducing the number of sensors to a great extent as shown in Figure 8. The difference between thermal gradient attraction and geometric center is that thermal gradient attraction method gives relatively poor results compared with geometric center method, while requiring a fewer number of thermal sensors.

**Table 3:** **Hot spot temperature error and corresponding number of sensors using different sensor allocation and placement approaches**

Approach	Allowable error %	Average error %	Number of sensors
**IKmC**	5	4.24	4
	4	3.33	13
	3	2.64	24
	2	1.32	32
	1	0.53	46
**GC**	5	4.73	4
	4	3.55	12
	3	2.78	20
	2	1.61	27
	1	0.66	39
**TGA**	5	4.80	2
	4	3.93	8
	3	2.97	15
	2	1.87	24
	1	0.76	35

**Figure 10:**
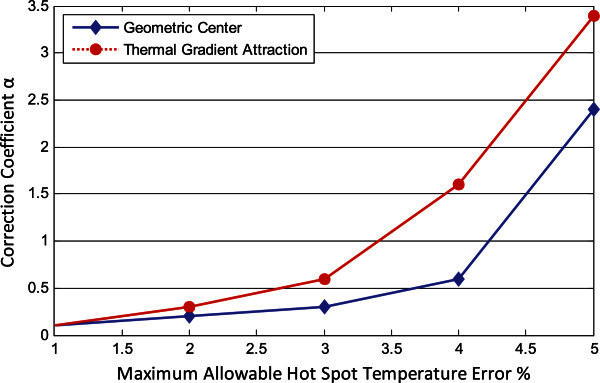
**Optimal values of correction coefficient using various sensor allocation and placement methods.**

The optimal value of correction coefficient *α* as a function of the maximum allowable hot spot temperature error is given in Figure 10. Note that the relationship between them is an increasing function. The reason is that when the maximum allowable hot spot temperature error increases, the non-spatial distance correspondingly increases and as the relationship between the threshold of non-spatial distance and correction coefficient is linear as defined in Sensor Allocation Algorithm section, the value of correction coefficient also increases. In addition, the optimal values of correction coefficient of thermal gradient attraction method are larger than those of geometric center method, which illuminates that thermal gradient attraction method achieves the hot spot estimation error limit bounded by the corresponding maximum allowable hot spot temperature error even with larger threshold of non-spatial distance.

**Figure 11:**
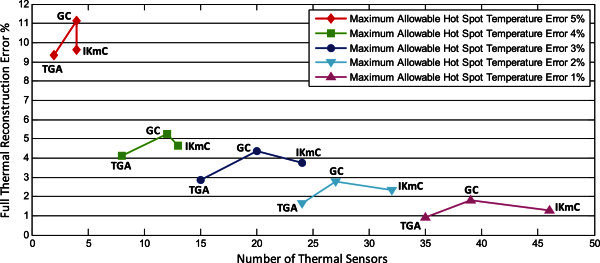
**Number of thermal sensors and full thermal reconstruction error as a function of thermal sensor allocation and placement methodology.**

The objective of our third set of experiments is to determine the full thermal reconstruction error while varying the maximum allowable hot spot temperature error accuracy from 1% to 5%. Figure 11 summarizes the errors in full thermal reconstruction and the corresponding allocation number of thermal sensors. It’s clear that thermal gradient attraction method gives the superior results: obtaining the least full thermal reconstruction error and requiring the fewest number of thermal sensors. The reason for the superior performance of the thermal gradient attraction strategy is that the sensor’s attraction towards steep thermal gradient is maximized. The difference between geometric center method and improved *k*-means clustering (Memik et al. 2008) is that geometric center method gives relatively poor results compared with improved *k*-means clustering (Memik et al. 2008), while requiring a fewer number of thermal sensors.

In conclusion, using our proposed thermal gradient attraction method, the allocation number of thermal sensors are 2, 8, 15, 24, 35, and the average absolute full thermal reconstruction errors are 9.34%, 4.12%, 2.88%, 1.65%, 0.91%, depending on different maximum allowable hot spot temperature error accuracy of 5%, 4%, 3%, 2%, 1%, respectively. These values confirm that our proposed thermal sensor allocation and placement techniques are capable of accurately characterizing the temperature of microprocessors, while requiring the fewest number of thermal sensors.

## Conclusion

In this paper, we have proposed systematic and effective techniques for determining the fewest number of thermal sensors and the optimal locations based on dual clustering algorithm in a complex microprocessor system. Our goal is to provide accurate thermal monitoring while maintaining a reasonable number of sensors. We first develop method based on dual clustering algorithm that can reduce the number of sensors to a great extent while satisfying an expected accuracy. Then we identify an optimal physical location for each sensor such that the sensor’s attraction towards steep thermal gradient is maximized.

The effectiveness of our techniques has been evaluated on a sophisticated experimental setup. Experimental results indicate the superiority of our techniques and confirm that our proposed thermal sensor allocation and placement techniques are capable of accurately characterizing the temperature of microprocessors, while requiring the fewest number of thermal sensors. The significance of our techniques will allow dynamic thermal management scheme to implement the accurate temperature monitoring with small number of embedded thermal sensors-a desirable property for microprocessors.

Our future work will focus on investigating the impact of calibration errors in the thermal sensor measurements (Zhang & Srivastava 2009; Zhang & Srivastava 2011) on the results of our proposed methods.
